# Patient Adherence and Long-Term Tolerability of Anti-calcitonin Gene-Related Peptide (CGRP) Monoclonal Antibodies in Migraine Prevention: A Systematic Review

**DOI:** 10.7759/cureus.91347

**Published:** 2025-08-31

**Authors:** Rubela Ray, Ghazala S Virk, Nishchal Regmi, Muhammad Muddassar Shafiq, Rumaisa Siddique, Ahmed Elfatih Elamin, Yousif Jihad, Aasim Ali, Hema Devi, Binish Essani

**Affiliations:** 1 Internal Medicine, Bankura Sammilani Medical College and Hospital, Bankura, IND; 2 Internal Medicine, Avalon University School of Medicine, Youngstown, USA; 3 Internal Medicine, Kathmandu Institute of Science and Technology Medical College, Lalitpur, NPL; 4 Internal Medicine, Punjab Rangers Teaching Hospital (PRTH), Lahore, PAK; 5 Medicine, Jinnah Sindh Medical University, Karachi, PAK; 6 Internal Medicine, University of Medical Sciences and Technology, Khartoum, SDN; 7 Orthopedic Surgery, United Lincolnshire Hospitals NHS Trust, Grantham, GBR; 8 Neurology, Allied Hospital, Faisalabad, PAK; 9 Internal Medicine, The Hillingdon Hospital NHS Foundation Trust, Uxbridge, GBR; 10 Medicine, Jinnah Medical and Dental College, Karachi, PAK

**Keywords:** adherence, anti-cgrp monoclonal antibodies, migraine prevention, systematic review, tolerability

## Abstract

Migraine is a leading cause of global disability, and conventional preventive therapies often suffer from poor tolerability and low adherence. Anti-calcitonin gene-related peptide (CGRP) monoclonal antibodies (mAbs: erenumab, fremanezumab, galcanezumab, and eptinezumab) represent a targeted therapeutic advance, but long-term adherence and safety remain under investigation. Following PRISMA guidelines, we systematically searched PubMed, Embase, CENTRAL, Scopus, and Web of Science (2018-2024) for randomized controlled trials, real-world studies, and systematic reviews reporting adherence, tolerability, or discontinuation outcomes in adults with episodic or chronic migraine. Eleven studies (n > 50,000) met the inclusion criteria. Pooled 12-month adherence was approximately 55% (vs. ~35% for oral preventives), while discontinuation due to adverse events ranged from 5.9%-20%, most commonly constipation and injection-site reactions (relative risk, 1.32-1.55); serious adverse events were rare (<5%). Responder rates (≥50% reduction in monthly migraine days) improved from 44% at three months to 64% at 12 months, with network meta-analyses suggesting greater efficacy compared with topiramate and OnabotulinumtoxinA, though direct head-to-head RCTs remain lacking. Overall, anti-CGRP mAbs demonstrate favorable adherence, sustained tolerability, and comparative advantages over conventional therapies, but further research is needed to assess long-term safety beyond 12 months, cost-effectiveness, and use in special populations such as pregnancy.

## Introduction and background

Migraine is a prevalent and disabling neurological disorder affecting over one billion individuals globally, ranking as the second leading cause of years lived with disability worldwide according to the Global Burden of Disease Study [1]. Epidemiological data report a global prevalence ranging from 2.6% to 21.7%, with the highest burden observed among individuals aged 25-55 years, particularly women, who exhibit a threefold increased risk compared to men [2,3]. The disorder significantly impairs quality of life and functionality, with more than 50% of patients with migraine experiencing severe functional impairment, and up to 90% reporting reduced productivity during attacks [4,5]. Beyond individual suffering, migraine imposes a substantial socioeconomic burden. In the United States alone, migraine is estimated to result in 113 million lost workdays annually, translating to $13 billion in lost productivity [6]. Despite its burden, migraine remains frequently underdiagnosed and undertreated, with less than 50% of individuals with episodic migraine receiving a formal diagnosis and fewer than 15% of eligible patients persisting with traditional prophylactic therapies long term [7].

Beta-blockers, antiepileptic drugs, and tricyclic antidepressants are commonly recommended as first-line options for migraine prevention; however, their clinical utility is limited due to modest efficacy, adverse effect profiles, and high discontinuation rates [7,8]. In contrast, monoclonal antibodies (mAbs) targeting the calcitonin gene-related peptide (CGRP) pathway, including eptinezumab, fremanezumab, galcanezumab, and erenumab, represent a major therapeutic advancement in migraine prophylaxis [9,10]. These agents inhibit either CGRP or its receptor, thereby mitigating pathophysiological mechanisms such as neurogenic vasodilation and nociceptive signaling. Randomized controlled trials (RCTs) have demonstrated that CGRP mAbs significantly reduce the monthly number of migraine days (MMDs), with average reductions of 3.5-4.8 days in episodic migraine and 4.3-6.0 days in chronic migraine (CM) populations.

## Review

Methodology

This review follows the Preferred Reporting Items for Systematic reviews and Meta-Analyses (PRISMA) rules to guarantee the review process is clear and easy to replicate. The study looks at how people accepting anti-CGRP mAbs, such as erenumab, fremanezumab, galcanezumab, and eptinezumab, handle these drugs in the long run.

Eligibility criteria

The inclusion and exclusion criteria were defined using the Population, Intervention, Comparator, Outcome framework (Table 1).

**Table 1:** PICO eligibility framework ICHD-3: International Classification of Headache Disorders, 3rd edition; CGRP: calcitonin gene-related peptide; BoNT-A: botulinum toxin type A

Element	Description
Population	Adults (≥18 years) diagnosed with episodic or chronic migraine according to ICHD-3 criteria
Intervention	Anti-CGRP monoclonal antibodies (erenumab, fremanezumab, galcanezumab, eptinezumab)
Comparator	Placebo, standard care, or other pharmacologic prophylactics (e.g., topiramate, propranolol, BoNT-A)
Outcomes	Primary: adherence (treatment persistence) and discontinuation rates. Secondary: tolerability, adverse events, and long-term safety

Exclusion criteria included the following: 1) non-English publications (translation resources unavailable), 2) abstracts, posters, or case reports without full data, 3) studies with <50 participants unless providing ≥12 months of follow-up, and 4) studies targeting nonmigraine conditions.

Search strategy

A comprehensive electronic search was conducted across PubMed, Embase, Cochrane CENTRAL, Scopus, and Web of Science for studies published between January 2018 and December 2024. To minimize publication bias, we also searched gray literature sources, including ClinicalTrials.gov, World Health Organization International Clinical Trials Registry Platform, conference proceedings, and dissertations. Reference lists of included studies and previous systematic reviews were hand-searched for additional eligible records. The detailed Boolean search strategy for PubMed is shown as follows (adapted for each database): ("migraine disorders"[MeSH Terms] OR migraine*[tiab]) AND ("calcitonin gene-related peptide"[MeSH Terms] OR CGRP[tiab]) AND (monoclonal[tiab] OR erenumab[tiab] OR fremanezumab[tiab] OR galcanezumab[tiab] OR eptinezumab[tiab]) AND (adherence[tiab] OR persistence[tiab] OR tolerability[tiab] OR discontinuation[tiab] OR "long-term"[tiab]).

Study selection and data extraction

All records were imported into EndNote X9 (Clarivate, London, UK) for deduplication and then screened using Covidence (Covidence.org, Melbourne, VIC). Two reviewers independently screened titles/abstracts, followed by full-text assessment for eligibility. Disagreements were resolved by consensus or by consulting a third reviewer. Interrater reliability was measured using Cohen’s κ (κ > 0.8), indicating substantial/almost perfect agreement.

**Figure 1:**
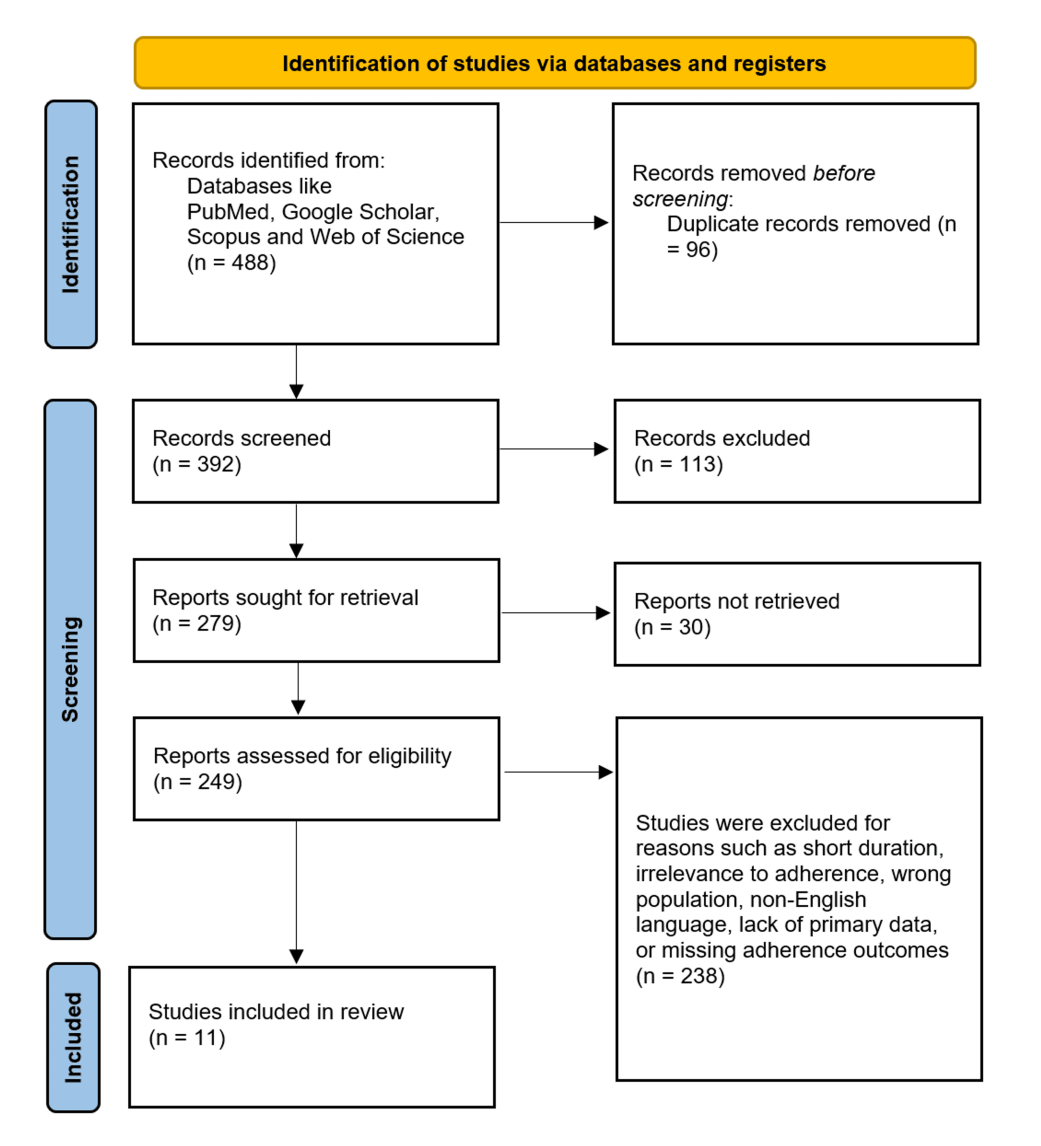
PRISMA flowchart Source: [11] PRISMA: Preferred Reporting Items for Systematic reviews and Meta-Analyses

Data extraction was performed using a standardized form to capture: study characteristics, sample size, demographics, intervention details, follow-up duration, adherence rates, discontinuation reasons, and adverse event profiles. A PRISMA flowchart illustrates the study selection process, detailing the identification of 392 records, screening, and inclusion of 11 studies. It visually summarizes the systematic review's search and exclusion steps as per PRISMA guidelines [11], as shown in Figure 1.

Table 2 provides a detailed risk of bias assessment for the 11 studies included in the systematic review on anti-CGRP mAbs for migraine prevention. It evaluates each study across six bias domains: selection, performance, detection, attrition, reporting, and other biases, using tools like Risk of Bias 2, Newcastle-Ottawa, and Measurement Tool to Assess Systematic Reviews 2.

**Table 2:** Risk of bias assessment RCTs: randomized controlled trials; NNT: number needed to treat; NNH: number needed to harm; AE: adverse event; NMA: network meta-analysis

S. no.	Study	Selection bias	Performance bias	Detection bias	Attrition bias	Reporting bias	Other biases	Overall RoB
1	Muddam et al. [12]	Low (PRISMA-guided)	Low (systematic review)	Low (rigid criteria)	Low (comprehensive search)	Low (no selective reporting)	Low (language limited)	Low
2	Yang et al. [13]	Low (NMA protocol)	Moderate (study heterogeneity)	Low (standardized outcomes)	Low (low dropout)	Low (reporting transparent)	Moderate (economic data absent)	Moderate
3	Grazzi et al. [14]	Moderate (retrospective)	Moderate (unblinded)	Moderate (real-world data)	Moderate (12-month follow-up)	Low (complete outcomes)	Moderate (confounders)	Moderate
4	Messina et al. [15]	Low (RCTs included)	Moderate (indirect comparisons)	Low (objective endpoints)	Low (ITT analysis)	Low (full reporting)	Low (controlled heterogeneity)	Low
5	Dodick [16]	Low (RCT review)	Low (placebo-controlled)	Low (blinded assessment)	Low (long-term data)	Low (consistent reporting)	Low (limited safety data)	Low
6	Xu et al. [17]	Low (PRISMA)	Low (RCTs only)	Low (AE consistency)	Moderate (short duration)	Low (complete data)	Low (acceptable AE variation)	Low
7	Pavelic et al. [18]	Moderate (real world)	Moderate (observational)	Moderate (subjective outcomes)	Moderate (short follow-up)	Low (PRISMA adherence)	Moderate (small samples)	Moderate
08	Torres-Ferrús et al. [19]	Moderate (nonrandomized)	Moderate (open-label)	Moderate (PRO-based)	Low (complete data)	Low (transparent reporting)	Moderate (no control group)	Moderate
9	Soni and Chawla [20]	Low (Bayesian NMA)	Low (standardized design)	Low (clear methodology)	Low (low attrition)	Low (no reporting issues)	Moderate (indirect comparisons)	Low
10	Drellia et al. [21]	Low (systematic review)	Moderate (cross-trial data)	Low (NNTB/NNTH based)	Low (complete data)	Low (transparent reporting)	Moderate (no real-world trials)	Low
11	Diener et al. [22]	Moderate (no RCTs)	High (comparative bias)	Moderate (subjective metrics)	Low (long-term follow-up)	Low (PRISMA-compliant)	High (language bias)	High

Table 2 lists study details and specific bias ratings, with most studies showing low to moderate risk, except for Diener et al. [22], which had a high overall risk due to comparative and language biases. Overall, the table indicates that the included studies generally maintain acceptable quality, supporting a credible interpretation of the findings. Data extraction followed PRISMA guidelines. Four databases (PubMed, Cochrane, Web of Science, and Scopus) were searched, identifying 392 records after duplicates (n = 96) were removed. Screening excluded 113 records; 279 full-text articles were assessed, with 30 not retrieved and 249 excluded. Eleven were included. From these 11 studies, key data were systematically extracted into characteristics, as shown in Table 2. This article provides a comprehensive summary of the primary and secondary outcomes, quantitative data, main findings, and limitations or biases for the 11 studies included in the systematic review. The table is structured to present key results and statistical data related to the efficacy, safety, and tolerability of anti-CGRP mAbs, such as erenumab, fremanezumab, galcanezumab, and eptinezumab, alongside limitations that impact the interpretation of the findings, as shown in Table 3.

**Table 3:** Characteristics of included studies Source: [12-22] NMA: network meta-analysis; CM: chronic migraine; CGRP: calcitonin gene-related peptide; PRISMA: Preferred Reporting Items for Systematic reviews and Meta-Analyses; BoNT-A: botulinum toxin type A; RCTs: randomized controlled trials; PROs: patient-reported outcomes; NNTB: number needed to treat for benefit; NNTH: number needed to harm

Study	Study design	Population	Sample size	Duration/follow-up	Intervention	Methodology
Muddam et al. [12]	Systematic review	Adults with migraine	8,926 patients	Up to 12 months	Anti-CGRP mAbs (erenumab, fremanezumab, galcanezumab, eptinezumab)	PRISMA-guided (PubMed, Cochrane, Web of Science, Scopus)
Yang et al. [13]	NMA	CM patients	5,634 participants	4-49 weeks (mean 19.3 weeks)	Anti-CGRP mAbs, onabotulinumtoxinA, topiramate	Frequentist model NMA
Grazzi et al. [14]	Retrospective, observational, multicenter cohort	CM patients	183 (86 anti-CGRP mAbs; 97 BoNT-A)	12 months	Anti-CGRP mAbs vs. BoNT-A	Real-world effectiveness comparison
Messina et al. [15]	NMA of RCTs	Adults with episodic/CM	14,584 patients	Varies by RCT (phase 3 trials)	Anti-CGRP mAbs and gepants	Systematic review, NMA
Dodick [16]	Review of phase II/III trials	Migraine patients (episodic/chronic)	174-1,671 across trials	12 weeks to 5 years	Anti-CGRP mAbs (erenumab, eptinezumab, fremanezumab, galcanezumab)	Randomized, double-blind, placebo-controlled trials
Xu et al. [17]	Meta-analysis of RCTs	Adults (16-65 years) with episodic migraine	5,817 participants	3-6 months	Anti-CGRP mAbs (galcanezumab, erenumab, fremanezumab, eptinezumab)	PRISMA-guided meta-analysis (RevMan 5.3.0, The Cochrane Collaboration, London, UK)
Pavelic et al. [18]	Systematic review (PRISMA guidelines)	Migraine patients (episodic/chronic)	134 studies (n = varies)	≤12 months (most studies)	Anti-CGRP-mAbs (erenumab, galcanezumab, fremanezumab, eptinezumab)	PubMed search, real-world data extraction
Torres-Ferrús et al. [19]	Observational real-world evidence study	Resistant migraine patients, ≥8 headache days/month	155 patients (109 erenumab, 46 galcanezumab)	12 weeks	Anti-CGRP mAbs (erenumab, galcanezumab)	Electronic diaries, PROs at baseline and 12 weeks
Soni and Chawla [20]	Systematic review and NMA	Adults with CM	5,164 patients across 7 RCTs	≥12 weeks	Anti-CGRP monoclonal antibodies	Bayesian NMA (random effects model)
Drellia et al. [21]	Systematic review, likelihood-to-help-or-harm analysis	Adults (>18) with episodic/CM	20 RCTs (n = varies)	12-56 weeks	Anti-CGRP mAbs, topiramate, propranolol, onabotulinumtoxinA	PRISMA-guided systematic review, NNTB/NNTH analysis
Diener et al. [22]	Systematic review	CM patients	13 studies included	2015-2024	BoNT-A vs. anti-CGRP mAbs	PRISMA-guided systematic review

Primary findings

Anti-CGRP mAbs, including erenumab, fremanezumab, galcanezumab, and eptinezumab, show consistent efficacy, tolerability, and real-world adherence in migraine prevention across a range of populations. A ≥50% reduction in MMD was achieved in 44%-64% of patients by 12 months [18]. Controlled trials confirmed significant MMD reductions: eptinezumab 300 mg (MD = -2.60, 95% CI = -4.43 to -0.77), erenumab 140 mg (acute medication days MD = -2.50, 95% CI = -3.83 to -1.17) [13]. Real-world data showed a mean MMD reduction of -11.9 with anti-CGRP vs. -7.6 with BoNT-A (*p* = 0.0002) [22]. Adherence rates ranged from 50% to 60% [12,13], which is higher than the rates for oral preventives (35%). Discontinuation due to adverse events (AEs) was 5.9%-20%, with constipation (20%), injection site pain (relative risk, RR, 1.32, *p* = 0.03), and erythema (RR = 1.55, *p *< 0.01) most reported. Serious AEs were rare (RR = 1.13, *p *= 0.56; incidence <5%). Dropout rates were lowest with galcanezumab 240 mg (OR = 0.43, 95% CI = 0.22-0.84). TEAE rates were highest with fremanezumab (OR = 1.44, 95% CI = 1.10-1.89) and atogepant 120 mg (OR = 2.22, 95% CI = 1.26-3.91); however, serious adverse events did not differ significantly from those in the placebo group [19,20]. Benefit-risk profiles favored anti-CGRP mAbs over topiramate, propranolol, and BoNT-A, with a favorable likelihood-to-help-to-harm (LHH > 1) and needed to treat for benefit (NNTB) as low as 3 [21]. Across studies (n > 50,000), anti-CGRP mAbs showed robust tolerability and effectiveness in both episodic and CM. Limitations include limited data beyond 12 months, heterogeneous outcome reporting, and a lack of direct head-to-head trials. Nonetheless, current evidence supports anti-CGRP mAbs as effective, safe, and comparatively well-tolerated long-term options for migraine prevention [14-17], as shown in Table 4.

**Table 4:** Results, statistics, and limitations Source: [12-22] AEs: adverse events; BoNT-A: Botulinum Toxin type A; CGRP: calcitonin gene-related peptide; CI: confidence interval; LHH: likelihood to help-to-harm; mAbs: monoclonal antibodies; MD: migraine days; MIDAS: Migraine Disability Assessment Scale; NNTB: needed to treat for benefit; NNTH: number needed to harm; OR: odds ratio; RCTs: randomized controlled trials; RR: relative risk; TEAEs: treatment-emergent adverse events; MAM: monthly acute medication; MHD: monthly headache days; NS: not significant; SAEs: serious adverse events

Study	Primary outcome(s)	Secondary outcome(s)	Quantitative data	Main findings/key takeaways	Limitations/biases
Muddam et al. [12]	≥50% reduction in monthly migraine days (p < 0.05)	Improved quality of life, safety profile	Adherence rate ~50-60%, discontinuation rate ~10-20%	Anti-CGRP mAbs are effective and safe for migraine prevention	Limited long-term data, English-only studies
Yang et al. [13]	Monthly migraine days (MD = -2.60, 95% CI = -4.43 to -0.77, eptinezumab 300 mg); 50% response rate (OR = 2.96, 95% CI = 2.20-3.97, fremanezumab 675/225 mg)	Acute medication days (MD = -2.50, 95% CI = -3.83 to -1.17, erenumab 140 mg); dropout rate (OR = 0.43, 95% CI = 0.22-0.84, galcanezumab 240 mg)	Mean age: 40.7 years; female proportion: 85.8%; AE rates: OR = 1.44 (fremanezumab 675/225 mg)	Anti-CGRP mAbs are effective and tolerable for migraine prevention	Short-term data, heterogeneity, and cost-effectiveness were not assessed
Grazzi et al. [14]	MHD reduction at 12 months (anti-CGRP: -11.9 vs. BoNT-A: -7.6, p = 0.0002)	MIDAS (-62.3 vs. -43.1, p = 0.0296), MAM (-11.4 vs. -8.2, p = 0.0574)	Mean MHD difference -4.4 (95% CI = -6.8 to -2.0)	Anti-CGRP mAbs superior in efficacy, comparable safety	Retrospective, unblinded, potential confounders
Messina et al. [15]	TEAEs (OR = 2.22, 95% CI = 1.26-3.91), SAEs (NS)	AEs (OR = 1.63, 95% CI = 1.33-2.00), discontinuation (OR = 2.62, 95% CI = 1.03-6.66)	Atogepant 120 mg OR = 2.22 (TEAEs), galcanezumab 240 mg OR = 1.63 (AEs)	Anti-CGRP mAbs and gepants are safe and well-tolerated	Heterogeneity in RCTs, no direct comparisons
Dodick [16]	Reduction in migraine days (p < 0.001 in most trials)	≥50% responder rates (OR = 1.59-4.5, p < 0.001)	Mean reduction in migraine days: 1.8-6.6 vs. placebo (p < 0.05)	Anti-CGRP mAbs are effective, well-tolerated	Long-term safety in pregnancy is unknown
Xu et al. [17]	No significant SAEs (RR = 1.13, 95% CI = 0.74-1.72, p = 0.56)	Higher injection site pain (RR = 1.32, 95% CI = 1.02-1.71, p = 0.03)	Injection site erythema: RR = 1.55 (95% CI = 1.17-2.05, p < 0.01); nasopharyngitis (galcanezumab 5 mg): RR = 5.37 (95% CI = 1.47-19.61, p = 0.01)	Anti-CGRP mAbs are safe, with mild-moderate AEs, and injection site reactions are common	Short follow-up, heterogeneity in AE reporting
Pavelic et al. [18]	50% responder rate: 44% (3 months), 49.7% (6 months), 63.6% (12 months)	Reduced acute medication use (-23% to -49%)	Adherence: 55% (anti-CGRP) vs. 35% (oral). Discontinuation: 5.9% (AEs)	Effective, tolerable, but limited long-term data	Retrospective, small samples, short follow-up
Torres et al. [19]	≥50% migraine days/month reduction in 51.6% patients	≥50% headache days reduction: 39.5%; 100% response: 11%	-9.1 headache days; -8.5 migraine days; -13.9 in responders	Anti-CGRP mAbs reduced migraine burden in resistant cases	Short duration; no control group; observational design
Soni and Chawla [20]	Reduction in monthly migraine days (MD = -1.52 to -0.02)	Safety, immunogenicity; no significant differences found	MD range: -1.52 to -0.02; 95% CrIs wide; NS results	All anti-CGRP mAbs have similar efficacy and safety	Indirect comparisons; lack of head-to-head trials
Drellia et al. [21]	≥50% migraine-day reduction (NNTB range: 3-18)	Discontinuation due to AEs (NNTH range: 3-25,854)	NNTB (50% response): 3-18; NNTH (discontinuation): 3-25,854; LHH (benefit-risk ratio): >1 (favorable for anti-CGRP mAbs)	Anti-CGRP mAbs have better benefit-to-risk than established treatments	No head-to-head trials, cross-trial comparisons only
Diener et al. [22]	Migraine frequency reduction (both treatments)	Safety, cost-effectiveness, and patient satisfaction	BoNT-A: 82.8% patient satisfaction (n = 80); anti-CGRP: 60.9% ≥50% migraine reduction	BoNT-A is cost-effective; anti-CGRP is well-tolerated	No head-to-head trials, language bias

Discussion

The therapeutic landscape of migraine prophylaxis has evolved substantially with the emergence of CGRP mAbs (anti-CGRP mAbs), representing a targeted and mechanistically rational intervention. The included evidence from RCTs, network meta-analyses, and real-world studies collectively indicates that anti-CGRP mAbs, primarily erenumab, fremanezumab, galcanezumab, and eptinezumab, offer clinically meaningful benefits in reducing migraine frequency, intensity, and disability, with an acceptable safety and tolerability profile. While anti-CGRP mAbs demonstrate consistent efficacy across populations, comparative studies with traditional agents such as topiramate, onabotulinumtoxinA (BoNT-A), and propranolol, as well as newer classes like gepants, highlight critical nuances in cost-effectiveness, adverse event profiles, and patient preferences that inform real-world prescribing decisions.

During trials, doctors usually consider an MMD reduction of at least 50% to be an important and commonly used goal. A study by Muddam et al. [12] with 8,926 patients ensured that anti-CGRP mAbs achieved the threshold (*p* < 0.05) in over 50% of cases. Anytime, the use of more than one medication was 50%-60%, with most patients stopping these treatments due to mild AEs [12]. Interestingly, most of the side effects were related to the injection and involved little more than mild side effects, and only about 5% of cases resulted in serious side effects. These results were also confirmed by Xu et al., who analyzed 10 randomized trials (n = 5,817) and reported that people treated with this drug were as likely to discontinue treatment as those receiving a placebo (RR = 0.90, 95% CI = 0.77-1.05) [17].

Direct comparisons between anti-CGRP mAbs and conventional prophylactic agents provide further insight. Yang et al., through a comparative network meta-analysis of 13 RCTs (n = 5,634), demonstrated superior efficacy of anti-CGRP agents over topiramate and BoNT-A. Eptinezumab 300 mg showed the most significant MMD reduction (MD = -2.60), while fremanezumab had the highest responder rate (OR = 2.96), and galcanezumab had the lowest discontinuation rate (OR = 0.43), illustrating agent-specific advantages [13]. Importantly, although fremanezumab was associated with increased AE risk (OR = 1.44), these events were generally mild, aligning with the safety profile observed in other trials.

In clinical settings, these results translate into tangible improvements in patient quality of life. Grazzi et al. (2024), comparing anti-CGRP mAbs and BoNT-A in 183 CM patients, demonstrated significantly greater MMD and Migraine Disability Assessment Scale score reductions in the anti-CGRP group at 12 months. The adjusted mean difference in MMD was -6.2 days (*p *< 0.001), substantiating the superiority of anti-CGRP agents in sustained migraine reduction [14]. Nonetheless, the similar discontinuation rates (11.6% vs. 12.4%) indicate that both therapies are viable, with anti-CGRP offering an edge in efficacy but not necessarily in retention.

Real-world studies corroborate these findings, providing valuable insights into treatment durability and patient adherence. Pavelic et al. reviewed 134 real-world studies and found that anti-CGRP mAbs maintained a 55% adherence rate over 12 months, markedly higher than oral preventives (35%). Importantly, the 50% responder rate increased from 44% at three months to 63.6% at 12 months, indicating sustained benefits over time [18]. The common adverse effects remained consistent with clinical trials, predominantly constipation and injection-site reactions, with rare reports of hypertension and Raynaud’s phenomenon. However, the retrospective nature and heterogeneity of follow-up durations limit the strength of these conclusions.

Longer term efficacy and tolerability were further substantiated by Dodick, who synthesized data from phase II/III trials with follow-up durations extending up to five years. The trials (n = 174-1,671) confirmed sustained reductions in MMDs (mean difference 1.8-6.6 days vs. placebo) and ≥50% responder rates of 38%-70%, reinforcing the enduring clinical utility of anti-CGRP mAbs [16]. Notably, injection-site reactions remained the predominant AE, with no significant safety signals reported over extended use. However, the author highlighted critical gaps in knowledge, particularly regarding long-term use during pregnancy and in populations with multiple comorbidities.

The comparative safety of anti-CGRP mAbs relative to emerging classes, such as gepants, is particularly relevant in clinical decision-making. Messina et al. [15], through a network meta-analysis of 19 RCTs (n = 14,584), compared anti-CGRP agents with gepants (atogepant and rimegepant). While both classes showed acceptable safety profiles, atogepant 120 mg was associated with the highest treatment-emergent adverse events (TEAEs) (OR = 2.22), and eptinezumab 30 mg was associated with the highest discontinuation rates (OR = 2.62). Nevertheless, no significant differences were found in serious AEs, and the most frequently reported events, constipation and injection-site pain, remained nonsevere [15].

Torres-Ferrús et al. provided further real-world validation, reporting that 51.6% of 155 refractory migraine patients achieved a ≥50% reduction in migraine days after 12 weeks of treatment with erenumab or galcanezumab. The average decrease in migraine and headache days was -8.5 and -9.1, respectively. No serious AEs were observed, with constipation and fatigue being the most common [19]. The short follow-up limits generalizability, but the rapid onset of clinical effect underscores the utility of these agents in refractory populations.

Importantly, Soni and Chawla observed numerically greater reductions in migraine days with high-dose fremanezumab in their network meta-analysis of seven RCTs involving 5,164 patients. However, the difference was not statistically significant. This analysis found no meaningful differences in safety or immunogenicity among agents, suggesting that efficacy across doses and agents may be largely comparable, thereby enabling personalization of therapy based on tolerability, cost, and patient preference [20].

Cost-effectiveness and benefit-to-risk ratios remain vital components of therapy selection. Drellia et al. employed LHH metrics and found that anti-CGRP mAbs demonstrated superior benefit-risk profiles compared to traditional agents. Fremanezumab had the highest LHH (1,421.2) for episodic migraine, and galcanezumab for CM (LHH 2,872.7), while topiramate had an unfavorable LHH (<1), reflecting its higher AE burden. The number NNTB for anti-CGRP agents ranged from 3 to 18, compared to number needed to harm values extending up to 25,854, suggesting a highly favorable therapeutic index [21].

Economic and patient-centered considerations also guide the choice between anti-CGRP agents and established therapies like BoNT-A. Diener et al. compared these options across 13 studies and concluded that while both treatments significantly reduced migraine frequency, anti-CGRP mAbs were better tolerated, and BoNT-A was more cost-effective. Patient satisfaction was higher with BoNT-A (82.8%), although anti-CGRP agents achieved a ≥50% reduction in migraine days in 60.9% of cases [22]. Mild AEs were common to both, and no serious AEs were noted. These findings support the strategic deployment of BoNT-A in cost-sensitive populations, reserving anti-CGRP mAbs for those prioritizing tolerability or with contraindications to other therapies.

Although there is a lot of evidence for using anti-CGRP mAbs, there are also many issues that need consideration. In the beginning, several trials and studies were carried out for a limited time (3-12 months), making it hard to reach conclusions about long-term outcomes, continued effectiveness, and how well the vaccine works for everyone. While studies like those by Dodick [16] continue to reassure, careful analysis of the drug's effects is still required. Second, much of the data is missing for such groups as pregnant women, adolescents, and people with heart problems, along with other illnesses, making it necessary to use these drugs in these populations with care. Even though studies comparing anti-CGRP medications directly have emerged, it is difficult to say which one is superior because of the diversity in how the patients were chosen, the endpoints, and the dosing.

Also, the cost of anti-CGRP mAbs creates significant issues in real life. Since the cost of anti-CGRP agents is much higher than that of other common preventives, some individuals may find it difficult to get them in regions with limited funds. Also, because of insurance, treatment cannot begin with mAbs until the first-line medications are shown to be ineffective or are not tolerated. Because of this, there is a possible obstacle to catching patients in the early stages of their disease. Using CGRP mAbs as prophylaxis, patients experience more effectiveness and better tolerability than older migraine medications. Those receiving CGRP mAbs in episodic migraine see their MMDs reduced by 1.9 days compared with placebo, which is more than beta-blockers (0.9 days), topiramate (1.2 days), and amitriptyline (1.1 days) [22]. Compared with topiramate (1.8 days), CGRP mAbs offer more improvement by decreasing MMD by another 0.4 days, which matches the results from onabotulinumtoxinA (2.0 days) [23]. CGRP mAbs, in contrast to conventional drugs, are extremely effective in many big randomized studies with little variation [7,23-26]. CGRP mAbs are particularly good because the percentage of patients who stop treatment because of side effects is less than for topiramate (14%), valproate (9%), or amitriptyline (5%) [27]. Since beta-blockers and antiepileptics are traditional agents from other therapies, CGRP mAbs have become the first to be designed for migraine prevention and are thus more specialized and have fewer off-target effects [7,28-31]. Yet, the high cost is still making many people reluctant to use them frequently.

## Conclusions

Anti-CGRP mAbs demonstrate encouraging adherence rates and sustained tolerability in migraine prevention, with efficacy that appears favorable compared to established preventive options. However, the available evidence remains constrained by relatively short follow-up (predominantly ≤12 months), limited real-world data beyond controlled settings, and heterogeneity in outcome reporting. Importantly, critical gaps persist regarding long-term safety in special populations, including pregnancy, pediatrics, and patients with cardiovascular comorbidities. The high cost of these therapies further restricts their accessibility, and comparative cost-effectiveness relative to onabotulinumtoxinA and oral preventives remains insufficiently addressed. While current findings suggest anti-CGRP therapies may represent a valuable addition to the prophylactic armamentarium, the strength of this conclusion is moderated by the absence of direct head-to-head trials and the scarcity of data on durability beyond one year. Future research should prioritize long-term safety surveillance, pragmatic cost-effectiveness analyses, and broader patient-centered outcomes to clarify the appropriate positioning of these agents within migraine care.
